# Editorial: Commercialization and industrialization of pharmacology of infectious diseases: 2022

**DOI:** 10.3389/fphar.2024.1438072

**Published:** 2024-06-19

**Authors:** Yanqin Huang, Sue C. Nang, Yu-Wei Lin, Fekade Sime

**Affiliations:** ^1^ Department of Pathology, Beth Israel Deaconess Medical Center, Boston, MA, United States; ^2^ Harvard Medical School, Boston, MA, United States; ^3^ Department of Microbiology, Biomedicine Discovery Institute, Monash University, Melbourne, VIC, Australia; ^4^ Monash Institute of Pharmaceutical Sciences, Monash University, Melbourne, VIC, Australia; ^5^ Malaya Translational and Clinical Pharmacometrics Group, Faculty of Pharmacy, Universiti Malaya, Kuala Lumpur, Malaysia; ^6^ Department of Pharmacy and Pharmacy Practice, Faculty of Pharmacy, Universiti Malaya, Kuala Lumpur, Malaysia; ^7^ Certara Drug Development Solutions, Certara, Radnor, PA, United States; ^8^ Centre for Clinical Research, The University of Queensland, Brisbane, QLD, Australia

**Keywords:** personalized antibiotic dosing, population pharmacokinetic (PPK), hollow fiber infection model, augmented renal clearance, lefamulin, etimicin, teicoplanin, cefoperazone/sulbactam

To ensure effectiveness, antibiotics must achieve therapeutic exposures at the target site of infection within the body. However, patients, particularly those who are critically ill, exhibit varying conditions that can impact drug absorption, distribution and elimination. When standard dosing regimens remain unadjusted, subtherapeutic antibiotic exposures may occur in significant proportion of critically ill patients, leading to compromised clinical outcomes and the risk of developing multidrug-resistant bacteria (Shi et al.). Thus, there is a growing need for personalized dosing adjustments in antibiotic therapy, guided by population pharmacokinetic (PPK) models. These models enable the application of personalized regimens to optimize treatment outcomes for patients through model-informed precision dosing.

The 2022 Research Topic on the Commercialization and Industrialization of Pharmacology in Infectious Diseases delves into advancements in pharmacological research related to infectious diseases, with the potential for translation into commercial products and large-scale industrial production. 1) Jacobsson et al. conducted a study evaluating the efficacy of lefamulin in treating gonorrhea using a hollow fiber infection model (Jacobsson et al.). 2) Ji et al. evaluated the efficacy of a new cefoperazone/sulbactam combination (3:1) for Enterobacteriaceae infection via model-informed drug development approaches (Ji et al.). 3) Chen et al. successfully developed a PPK model for teicoplanin, revealing that current standard doses may result in subtherapeutic exposures (Chen et al.). 4) Cui et al. explored two bioanalysis methods for detecting etimicin in human serum and urine (Cui et al., 2023). 5) Shi et al.’s review summarized personalized antibiotic dosage regimens for patients with augmented renal clearance (Shi et al.).

The rise and spread of antimicrobial resistance in *N. gonorrhoeae* pose a significant threat to the global management and treatment of gonorrhea. It is imperative to explore novel treatment options, accompanied by appropriate methods to pharmacodynamically assess the effectiveness and emergence of resistance to new drugs. Jacobsson et al. investigated the pharmacodynamics (PD) of lefamulin in treating *Neisseria gonorrhoeae* using a dynamic hollow fiber infection model (HFIM). Their study considered both the concentrations of free lefamulin in human plasma and estimations of free lefamulin concentrations in urogenital tissues. Given the lack of an ideal animal model for the obligate human pathogen *N. gonorrhoeae*, the use of a dynamic *in vitro* HFIM was essential. This model guided the optimal dosing required for effective *N. gonorrhoeae* eradication while suppressing the emergence of resistance. These findings underscore the necessity for a clinical study to fully elucidate the genuine clinical potential of lefamulin as a viable treatment option for uncomplicated gonorrhea.

Cefoperazone/sulbactam is a commonly prescribed antibiotic combination used to combat bacteria producing extended-spectrum beta-lactamases (ESBLs). Ji et al. evaluated the efficacy of a novel cefoperazone/sulbactam combination (3:1) for treating Enterobacteriaceae infections using model-informed drug development approaches. Their findings revealed that cefoperazone/sulbactam (3:1) did not demonstrate superior bactericidal activity to that of sulperazon [cefoperazone/sulbactam (2:1)]. Notably, poor antibacterial effect was observed for ESBL-producing and cefoperazone-resistant *E. coli* and *K. pneumoniae* strains. This study highlights the need for caution in proceeding with further clinical trials to mitigate the potential risks of not meeting expected treatment targets with cefoperazone/sulbactam combination (3:1).

Teicoplanin is widely utilized in treating infections caused by Gram-positive bacteria, including methicillin-resistant *Staphylococcus aureus* (MRSA). However, the effectiveness of current teicoplanin treatments faces challenges due to the relatively low and inconsistent concentrations achieved under standard dosage regimens. Chen et al. successfully developed a PPK model for teicoplanin in adult septic patients to optimize teicoplanin dosing regimens. Model-based simulations unveiled that the current standard doses may result in suboptimal exposure, suggesting that a single dose of at least 12 mg/kg might be necessary. Additionally, AUC_0-24_/MIC is proposed as the preferred *p*K/PD indicator for teicoplanin in scenarios where AUC estimation is not feasible. Additionally, routine monitoring of teicoplanin C_min_ on Day 4 and subsequent therapeutic drug monitoring at steady-state are recommended. This study marks the pioneering development of a PPK model for teicoplanin in ICU adult patients with sepsis. Furthermore, the study proposes a dose selection scheme for teicoplanin in septic patients with varying renal functions, considering both C_min_ and AUC_0-24_/MIC targets.

Etimicin, a fourth-generation aminoglycoside antibiotic, exhibits potent activity and low toxicity against both Gram-negative and Gram-positive bacterial infections. Despite its efficacy, the complete PK of etimicin in humans remain unclear. Cui et al. developed two liquid chromatography-tandem mass spectrometry (LC-MS/MS) bioanalytical methods devoid of ion-pairing reagents. These methods were validated for quantifying etimicin in human serum and urine (the first bioanalytical method for assessing etimicin in human urine samples via LC-MS/MS). Subsequently, these simple yet reliable methods were successfully applied in a dose-escalation, phase I clinical trial of etimicin in Chinese healthy volunteers. The trial involved intravenous administration of single and multiple doses. The resulting comprehensive pharmacokinetic data of etimicin in humans serve as a foundation for further exploration into breakpoint research and aid in promoting the rational use of antibiotics in clinical practice.

Augmented renal clearance (ARC) manifests as heightened renal function, often observed in 30%–65% of critically ill patients, despite maintaining normal serum creatinine levels. However, administering renally eliminated drugs to ARC patients using unadjusted standard dosing regimens frequently results in inadequate drug concentrations, leading to compromised clinical outcomes and the risk of multidrug-resistant bacteria. Shi et al. conducted a comprehensive review spanning from 2010 to 2022, utilizing the MEDLINE database to delve into pharmaceutical, pharmacokinetic, and pharmacodynamic research on ARC. Results showed that critically, ARC patients, particularly those in intensive care, necessitate tailored adjustments in antibiotic therapy, encompassing dosage, frequency, and route of administration (Kantasiripitak et al., 2020).

In conclusion, the variability in patient conditions, especially among critically ill patients, necessitates personalized dosing adjustments in antibiotic therapy to ensure therapeutic efficacy and prevent the emergence of resistance. Advanced pharmacometric models play a crucial role in guiding these tailored regimens and are being increasingly integrated with commercial Bayesian dosing software. Continued research and development in this field are essential to optimize treatment outcomes and enhance the overall management of infectious diseases.
